# Collagen Biosynthesis, Processing, and Maturation in Lung Ageing

**DOI:** 10.3389/fmed.2021.593874

**Published:** 2021-05-20

**Authors:** Ceylan Onursal, Elisabeth Dick, Ilias Angelidis, Herbert B. Schiller, Claudia A. Staab-Weijnitz

**Affiliations:** Comprehensive Pneumology Center, Institute of Lung Biology and Disease, Helmholtz-Zentrum München, Member of the German Center of Lung Research (DZL), Munich, Germany

**Keywords:** collagen, extracellular matrix, ageing, crosslinking, chronic lung disease, advanced-glycation end products, lysyl oxidase

## Abstract

In addition to providing a macromolecular scaffold, the extracellular matrix (ECM) is a critical regulator of cell function by virtue of specific physical, biochemical, and mechanical properties. Collagen is the main ECM component and hence plays an essential role in the pathogenesis and progression of chronic lung disease. It is well-established that many chronic lung diseases, e.g., chronic obstructive pulmonary disease (COPD) and idiopathic pulmonary fibrosis (IPF) primarily manifest in the elderly, suggesting increased susceptibility of the aged lung or accumulated alterations in lung structure over time that favour disease. Here, we review the main steps of collagen biosynthesis, processing, and turnover and summarise what is currently known about alterations upon lung ageing, including changes in collagen composition, modification, and crosslinking. Recent proteomic data on mouse lung ageing indicates that, while the ER-resident machinery of collagen biosynthesis, modification and triple helix formation appears largely unchanged, there are specific changes in levels of type IV and type VI as well as the two fibril-associated collagens with interrupted triple helices (FACIT), namely type XIV and type XVI collagens. In addition, levels of the extracellular collagen crosslinking enzyme lysyl oxidase are decreased, indicating less enzymatically mediated collagen crosslinking upon ageing. The latter contrasts with the ageing-associated increase in collagen crosslinking by advanced glycation endproducts (AGEs), a result of spontaneous reactions of protein amino groups with reactive carbonyls, e.g., from monosaccharides or reactive dicarbonyls like methylglyoxal. Given the slow turnover of extracellular collagen such modifications accumulate even more in ageing tissues. In summary, the collective evidence points mainly toward age-induced alterations in collagen composition and drastic changes in the molecular nature of collagen crosslinks. Future work addressing the consequences of these changes may provide important clues for prevention of lung disease and for lung bioengineering and ultimately pave the way to novel targeted approaches in lung regenerative medicine.

## Introduction

The extracellular matrix (ECM) is a highly dynamic non-cellular component of tissues that provides a complex structural network which serves as a scaffold for adherent and migrating cells. It is mainly composed of collagens, glycoproteins, proteoglycans, glycosaminoglycans, and several other components. By virtue of sequestered growth factors and ECM binding receptors at the surface of adherent cells, the ECM affects a plethora of cellular processes including cell proliferation, differentiation and migration and thus acts as a critical regulator of cell function (1, 2). It is well-established that the ECM plays an important role in the pathogenesis and progression of chronic lung disease (3, 4). Many chronic lung diseases, e.g., chronic obstructive pulmonary disease (COPD) and idiopathic pulmonary fibrosis (IPF) primarily manifest in the elderly, suggesting increased susceptibility of the aged lung or accumulated alterations in lung structure over time that favour disease. How the ECM changes during ageing, however, has not been comprehensively assessed or discussed.

Constituting between 30 and 70% of ECM protein in all tissue types, collagen is the main component of the ECM (5) and even forms the most abundant human protein class in general. Mutations and polymorphisms in genes encoding structural collagen chains as well as collagen biosynthetic proteins are associated with disease affecting the full age range. Some cause fatal congenital disorders leading to death very early in life, others result in premature or accelerated ageing in adolescents and adults, and yet others only become evident in the elderly where they lead to a high burden of multi-morbidity, reduced quality of life, and lower life expectancy (6). Probably the most widely known collagen-related disorders result in drastic bone and cartilage abnormalities, as e.g., brittle-bone *(osteogenesis imperfecta)* or Caffey disease, characterised by increased bone fragility or episodes of excessive bone formation, respectively (1). Other frequent effects of collagenopathies are skin alterations, visual defects and hearing loss, muscle weakness, vessel abnormalities and kidney disease (1).

Pulmonary manifestations of such collagen mutations and polymorphisms have received less attention, probably because the most severe lung abnormalities in such patients are caused by defects in chest formation and rib fractures, i.e., are of origin secondary to bone and cartilage defects (1, 6, 7). Nevertheless, altered collagen synthesis or turnover by other than genetic causes are frequent hallmarks of chronic lung disease and contribute considerably to disease progression, severity, morbidity, and mortality (3, 4). In lung cancer, for instance, dysregulated collagen expression and crosslinking appear to favour tumour progression by providing a permissive, pro-invasive, and pro-inflammatory environment (8). In pulmonary fibrosis, irrespective of disease aetiology, excessive collagen deposition in the alveolar space is the ultimate pathological feature leading to increasing dyspnoea and progressive lung function decline (9–14). In contrast, COPD/emphysema is characterised by increased degradation of ECM proteins by matrix metalloproteinases (MMPs) and neutrophil elastase, targeting primarily collagens and an unrelated major ECM protein, elastin, respectively (15).

Given that collagen is the most abundant protein type in the body, it is not surprising that collagen has been a subject of research for about 100 years by now. What is striking, however, is how little we know, nonetheless. The latest member of the collagen protein family, type XXVIII collagen encoded by COL28A1, has only been reported in the year 2006 (16). Similarly, new proteins acting in collagen biosynthesis and modification have only been discovered and characterised in the last two decades (17–21). Also, even though collagen is known to undergo excessive post-translational modification (PTM), both intra- and extracellularly, these PTMs have not been comprehensively mapped and the biological function of the majority of the PTMs remains unclear (22). Equally, it is poorly understood how collagen biosynthesis and turnover change during normal ageing and how such changes may affect the function of adherent cells, lung repair, susceptibility to disease, disease progression and comorbidities.

This review aims to draw attention to the complexity of collagen synthesis, processing, and degradation and the importance of these processes in lung ageing and chronic lung disease. To set the stage, we first provide an overview of collagen types and key steps of collagen biosynthesis, processing, and maturation. We then summarise what is known about collagen alterations in the ageing lung and what can be tentatively inferred from studies in other organs. Ultimately, we believe that a better understanding of these mechanisms may provide important clues for prevention of lung disease and for lung bioengineering and pave the way to novel targeted approaches in lung regenerative medicine.

## Collagen Types and Structure

**Figure 1:**
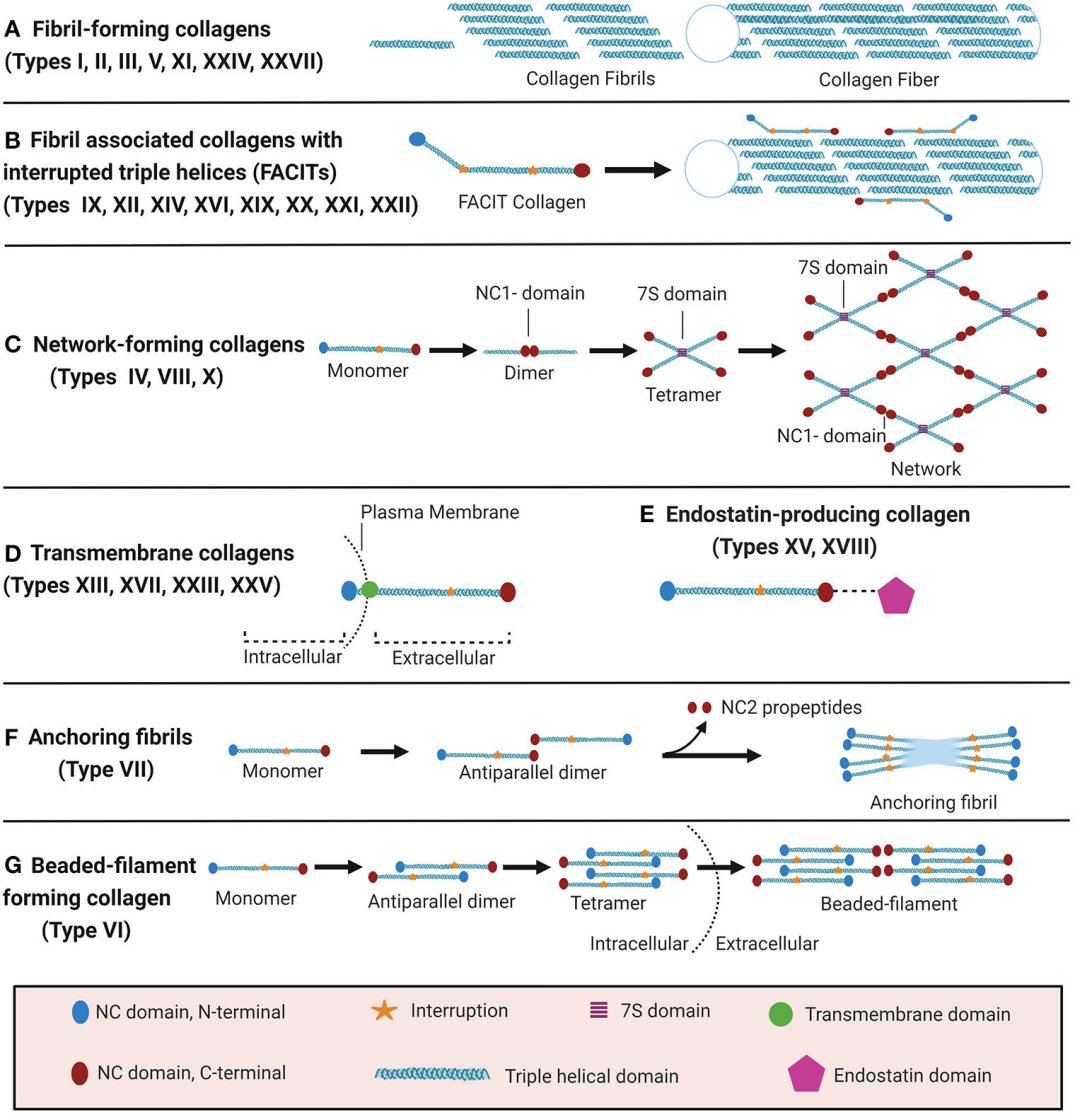
Classification of collagens based on supramolecular assembly. Schematic overview of the major forms of supramolecular assembly of collagen. For some non-collagenous (NC) domains, specific names are established in the literature and therefore specified in the figure: In the collagen IV network (C), the so-called 7S and NC1 domains represent critical nodes. In anchoring fibrils (F), the C-terminal domain which is cleaved off upon fibril formation is termed NC2. Figure was created with Biorender.com.

The collagen suprafamily comprises to date 28 known highly diverse collagen types in vertebrates, characterised by the presence of triple-helical collagenous domains. Collagens are divided into several subtypes depending on their domain structure and their macromolecular assembly (Figure 1): (A) Fibril-forming collagens (I, II, III, V, XI, XXIV, XXVII), (B) fibril-associated collagens with interrupted triple helices (FACITs, IX, XII, XIV, XVI, XIX, XX, XXI, XXII), (C) network-forming collagens (IV, VIII, X), (D) transmembrane collagens (XIII, XVII, XXIII, XXV), (E) endostatin-producing collagens or multiplexins (XV, XVIII), (F) anchoring fibrils (VII), and (G) beaded-filament-forming collagen (VI). Types XXVI and XXVIII do not fit well in any category (16, 23–25). Depending on the collagen type, collagens can assemble as homotrimers or heterotrimers. For instance, type III collagen is a homotrimer of three identical α1 chains (encoded by *COL3A1*), type I collagen typically assembles from two α1 chains (*COL1A1*) and one α2 chain (*COL1A2*) and type VI collagen from even three distinct chains (*COL6A1, COL6A2*, and *COL6A3* or *COL6A5* or *COL6A6*) (22, 26–28). Interestingly, altered chain stoichiometry has been described for several pathologies including fibrosis and may affect biochemical and biophysical properties of collagen (29–34).

### Collagen Domains and Macromolecular Assembly

The unifying feature of all collagens is the **triple-helical collagenous domain**, which is composed of three so-called α-chains consisting of amino acid repeats of (Gly-X-Y)_n_. The smallest amino acid glycine (Gly) can face the interior part of the triple helix while still allowing for a close association of the three chains. X and Y are often proline (Pro) or hydroxylated proline, 3-hydroxyproline (3-Hyp) or 4-hydroxyproline (4-Hyp), respectively (35, 36). While 4-Hyp in position Y of the Gly-X-Y repeat is frequently found in all collagen types and well-established as a major contributor to collagen thermodynamic stability (37–41), 3-Hyp has so far only been unambiguously detected in very few defined X positions of Gly-X-Y in collagen chains of type I, II, IV, and V, and 3-Hyp function is much less understood (42–46).

Frequent non-collagenous (NC) domains, e.g., in FACIT, beaded-filament forming and anchoring fibril collagens, are fibronectin type III, von Willebrand, thrombospondin (TSP) and Kunitz domains. The physiological function of these domains is incompletely understood. Fibronectin type III and von Willebrand domains seem to facilitate protein-protein interactions between collagens and other structural ECM molecules or growth factors in the extracellular space (47, 48). TSP domains mediate heparin and metal ion binding and may provide protection against blood clotting e.g., in subendothelial basement membranes (49, 50). The C-terminal Kunitz domains in type VI and VII collagens are cleaved off in the extracellular space (51–53). For type VI collagen, this cleavage product, endotrophin, stimulates tumour growth and angiogenesis, mediating many of the tumour-promoting effects of type VI collagen (54–56). Endotrophin is thus classified as a matricryptin or matrikine, a class of biologically active peptides derived from proteolytic processing of extracellular matrix proteins (57).

Even though all collagens are multi-domain proteins, the extent to which a collagen consists of collagenous and NC domains can differ drastically and the overall collagen domain architecture determines the macromolecular assembly (Figure 1). Type I collagen, like all **fibril-forming collagens**, contains a central uninterrupted collagenous domain as a major part of the polypeptide, flanked by relatively short NC domains, the N- and C-terminal telo- and propeptides (25). In contrast, **FACIT**s can contain <10% collagenous domains and associate to the surface of collagen fibrils but do not form such fibrils by themselves (23, 27). **Membrane collagens** comprise an NC cytoplasmic domain, a transmembrane domain, and extracellular repeats of triple-helical collagenous domains (27). Membrane collagen types XIII, XXIII, and XXV are also collectively referred to as membrane-associated collagens with interrupted triple helices (**MACIT**s) (58, 59). Notably, the C-terminal ectodomains of all membrane collagens can be proteolytically shed off the cell surface (23, 27, 60), and are classified as matrikines or matricryptins (27, 57, 61). Another collagen family that serves as a source for matricryptins are the **multiplexins** or endostatin-producing collagens. This family comprises collagen types XV and XVIII which generate restin and endostatin, respectively, upon proteolytic cleavage, both known for their potent antiangiogenic properties (24, 25, 27, 57, 58, 61, 62). The prototypical **network-forming collagen** type IV, a major basement membrane constituent, is encoded by in total 6 genes (*COL4A1*-*COL4A6*) the products of which form heterotrimers consisting of the chain combinations (α1)_2_α2(IV), α3α4α5(IV), or (α5)_2_α6 (IV) (23, 24, 27). The so-called 7S and NC1 domains are critical nodes in the collagen IV network and stabilised by covalent bonds, namely by LOXL2-mediated crosslinks between lysines in the 7S domain and the unique sulfilimine crosslink (-S=N-) in the NC1 domain (63–65). Also collagens VIII and X can assemble to form networks in tissues, but the molecular determinants have not yet been studied in similar detail (27). Type VII collagen is the major component of **anchoring fibrils** that are essential for the integrity of the dermo-epidermal junction in skin (24, 25, 27). It consists of two adjacent collagenous triple-helical domains which are flanked by a rather long (140 kDa) NC domain harbouring von Willebrand domains and fibronectin type III repeats at the N-terminus (NC1) and a much shorter (30 kDa) NC domain at the C-terminus (NC2) (27, 66). The initial formation of these anchoring fibrils from homotrimeric type VII collagen molecules involves antiparallel alignment of two collagen VII molecules at the level of their C-termini (NC2 domains), followed by enzymatic cleavage of the NC2 domain and stabilisation of the dimer by disulfide bonds (66). Finally, **beaded-filament-forming collagens** are strictly only represented by type VI collagen. Suprastructural assembly occurs as follows: Two heterotrimeric type VI collagen molecules dimerize in an antiparallel fashion *via* interaction of their central triple-helical domains. The protruding N-and C-terminal globular domains on both sides register in parallel with another dimer, forming a tetramer which is stabilised by disulphide bonds Finally, tetramers assemble end-to-end to generate the beaded-filaments (Figure 1) (23, 67). The collagen types XXVI and XXVIII are sometimes mentioned within the context of this class, even if they do not fit well in any category (16, 23–25). Type XXVIII collagen shares some sequence homology with type VI collagen (16). Type XXVI collagen, however, is uncharacteristically small for a collagen, comprises only two very short collagenous domains, and shares few similarities with any of the other described collagens (24).

## Collagen Biosynthesis

**Figure 2:**
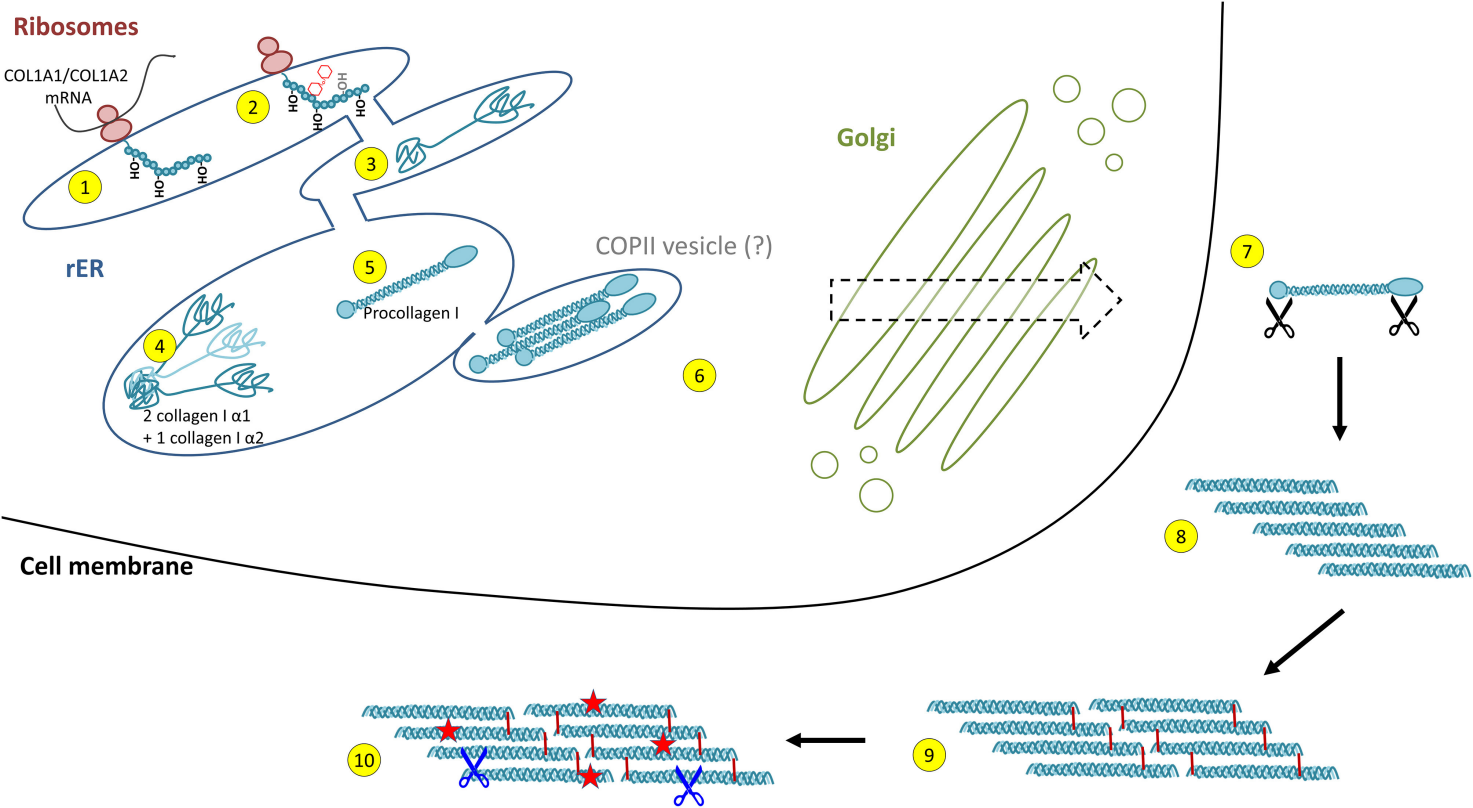
Intracellular collagen biosynthesis and extracellular maturation of collagen I. (1) Cotranslational prolyl-4- and lysyl-hydroxylation of the nascent collagen polypeptide chain in the rough endoplasmic reticulum (rER) is followed by (2) glycosylation and prolyl-3-hydroxylation and (3) folding of the C- and N-terminal propeptides. (4) For collagen I, two properly folded α1 chain C-propeptides assemble with one α2 chain C-propeptide, forming the triple helix nucleus. (5) Triple helix formation occurs in a zipper-like fashion and is dependent on peptidyl-prolyl isomerases and collagen chaperones. (6) Collagen triple helices are transported via the trans-Golgi network and finally secreted into the extracellular space. This was believed to occur *via* COPII vesicles, a concept that has been challenged recently. (7) In the extracellular space, propeptide cleavage involving at least three proteases (black scissors) triggers (8) auto-assembly of collagen fibrils. (9) Finally, fibrils are stabilised by crosslinking. (10) The mature collagen fibres are subject to insults (red stars) and degradation by extracellular proteases (blue scissors, see **Figure 4**).

Collagen biosynthesis is a highly complex process starting with transcription of collagen genes followed by translation and translocation of the nascent polypeptide chain to the rough ER (rER), co-translational modification and folding, trafficking across the Golgi network, secretion, and finally, extracellular processing and maturation (36, 68) (cf. Figure 2). This process is best described for type I collagen which will be used as an example for synthesis of a heterotrimeric fibril-forming collagen in the following.

### Translation and Co-translational Modification of the Nascent Polypeptides

**Figure 3:**
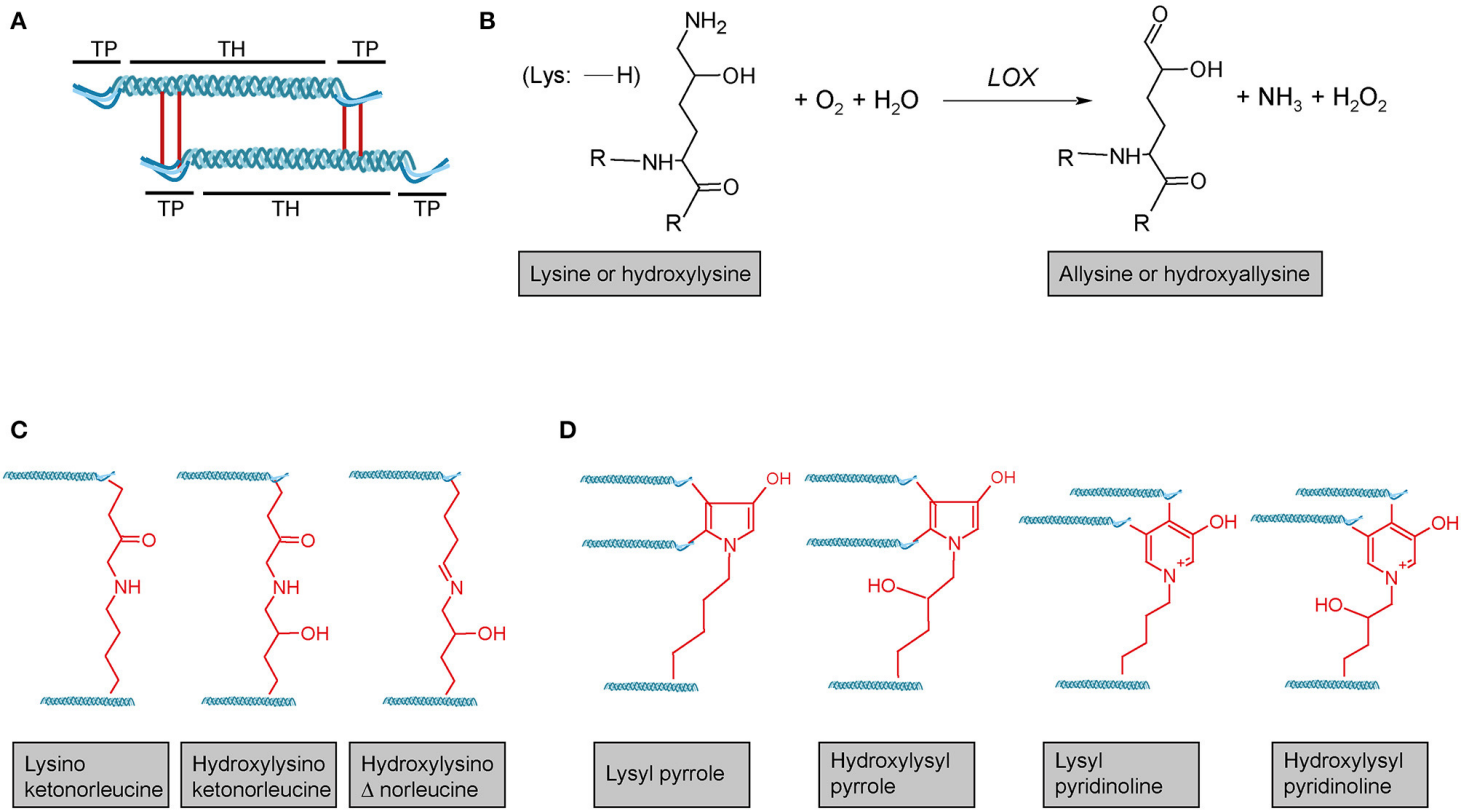
Lysyl oxidase-mediated crosslinking. **(A)** Schematic representation of telopeptide (TP) and triple-helical (TH) crosslink sites of two adjacent tropocollagen molecules in a collagen fibril. **(B)** Lysyl oxidase (LOX) enzymes initially catalyse the oxidative deamination of the ε-amino group of a lysine or hydroxylysine, yielding a highly reactive aldehyde group. This entails subsequent reactions with primarily other (hydroxy)lysines and rearrangements, ultimately resulting in different **(C)** divalent and **(D)** trivalent collagen crosslinks. R = continued polypeptide chain. Structures were generated using ACD/Chemsketch freeware.

Following transcription of *COL1A1* and *COL1A2* genes, the mRNA is translated into the polypeptide at the rER where the nascent polypeptide chain is extended into the lumen of the rER. Proper folding in the rER requires several enzymes and molecular chaperones essential for post-translational modifications (PTMs) and the formation of triple-helical procollagen molecules (36). First, numerous PTMs are introduced into the nascent unfolded polypeptide chain in a co-translational fashion. These include lysyl and prolyl hydroxylations and hydroxylysyl glycosylations, modifications that are mediated by several collagen-specific enzymes which exist in defined multiprotein complexes with other chaperones and protein folding catalysts [for an excellent review, see Ishikawa and Bächinger (36)]. Many of these PTMs are essential for proper stability, assembly and secretion of procollagen, as well as for the final supramolecular structure of these molecules (24, 25, 27, 36). Numerous enzymes catalysing PTMs have been identified. In vertebrates, prolyl-4-hydroxylation is mediated by one of three **prolyl-4-hydroxylases** (encoded by *P4HA1, P4HA2*, and *P4HA3*) which form complexes with protein disulphide isomerase (PDI). In the lung, at least one of these prolyl-4-hydroxylases, P4HA3, is upregulated in IPF and has been put forward as a potential drug target (69). Most members of the collagen **prolyl-3-hydroxylase** (P3H1-P3H4) family equally form multimeric complexes with other collagen-modifying proteins and chaperones. Of these, P3H1, P3H2, and P3H3 have been shown to exert prolyl-3-hydroxylase activity, while P3H4 (also termed SC65) forms a trimeric complex with P3H3 and lysyl hydroxylase 1 (LH1), but has no prolyl-3-hydroxylase activity of its own (19, 20). **Lysyl hydroxylases** comprise three enzymes termed LH1-LH3 or procollagen-lysine,2-oxoglutarate 5-dioxygenases 1 to 3 (PLOD1-PLOD3, e.g., Uniprot database) (36). LH1, as already mentioned above, exists in a complex with P3H3 and P3H4, and preferentially hydroxylates lysines in the triple-helical collagenous regions (70). The resulting 5-hydroxylysine residues may be subject to O-glycosylation by glycosyl transferases (36, 70, 71). In contrast, LH2, which associates with FKBP10 (FKBP65) is responsible for hydroxylation of lysines in the non-collagenous telopeptide regions of fibrillar collagens (70, 72). Notably, telopeptide lysines and hydroxylysine are subject to extracellular lysyl oxidase (LOX)-mediated crosslinking and the hydroxylation status of the involved lysines strongly affects nature and stability of the crosslink (for selected examples for crosslinks, see Figure 3) (36, 71–73). LH3 is known to hydroxylate lysines in type IV and V collagens but also exerts glycosyl transferase activity (36, 70, 71). Finally, in addition to LH3, two collagen **glycosyl transferases**, GLT25D1 and GLT25D2 mediate O-glycosylation of hydroxylysines *via* the 5-hydroxyl group. Here, hydroxylysines can be either O-glycosylated with a monosaccharide (β-d-galactopyranose, Gal) or a disaccharide (α-d-glucopyranosyl-(1->2)-β-d-galactopyranose, GlcGal), resulting in galactosylhydroxylysine (GHyl) or glucosylgalactosyl-hydroxylysine (GGHyl), respectively (36, 70, 71).

### Triple Helix Formation

Triple helix formation is preceded by folding of the N- and C-terminal propeptides and chain selection *via* the trimerization domains (36, 68, 74, 75), a process supported by a plethora of general ER-folding folding catalysts including Grp78 (BiP), Grp94, PDI, calreticulin, calnexin, and CypB (36). Following chain selection, triple helix formation is initiated and proceeds in a zipper-like fashion. Given the proportionally high number of proline residues in collagen, it is not surprising that one of the rate-limiting steps in triple helix formation is the *cis*-*trans* isomerization of proline residues catalysed by rER-resident **peptidyl-prolyl isomerases** (PPIases) (36, 68). Only the *trans-*proline conformation allows a linear prolongation of the triple helix (76). The PPIases FKBP10 (FKBP65), CypB as well as FKBP14 (FKBP22) appear to play critical roles in that context (36, 68, 77–79). In addition, **heat shock protein 47** (HSP47 or SERPINH1) functions as an important collagen-specific chaperone in collagen modification, triple helix formation, and export from the ER to the Golgi (36, 68, 80–82).

### Trafficking From the rER via the Trans-Golgi Network to the Extracellular Space

Procollagen secretion is dependent on coat protein complex II (COPII) vesicle-mediated transport from the rER to the Golgi. Typically, COPII vesicles are not bigger than 60–80 nm in diameter (83), while a completely folded and fairly rigid procollagen molecule can be up to 500 nm in length (84–88). Therefore, a long-held concept involved the transport of procollagen molecules *via* specialised, enlarged, COPII vesicles (85, 89–93). This concept, however, has been challenged recently: Analysis of endogenous and engineered GFP-tagged procollagen by live-cell imaging did not provide any evidence for dynamic large carrier vesicles between the ER and the Golgi but instead rather supports a model of direct interconnections between organelles or the presence of less well-characterised intermediate carriers (88, 94).

### Extracellular Processing and Maturation of Collagen

Extracellular processing and maturation of collagen depends very much on the collagen type and the nature of supramolecular assembly (cf. Figure 1) but is best described for type I collagen and other fibrillar collagens. Here, secretion is followed by the cleavage of the N- and C-terminal propeptides by specific procollagen proteinases, including bone morphogenetic protein 1 (BMP1), members of the ADAMTS protease family, and the more recently discovered meprins (23, 25, 27, 95). Notably, enzymatic cleavage of propeptides occurs for many non-fibrillar collagens, too, including the above-mentioned membrane collagens and multiplexins, also involves MMPs and cathepsins, and is the source of matrikines or matricryptins, collagen-derived fragments with diverse biological activities (57, 61). For fibrillar collagens, propeptide cleavage is followed by spontaneous, entropy-driven, quarter-staggered assembly of the tropocollagen molecules into fibrils (96, 97). Finally, intra- and intermolecular crosslinks, catalysed by enzymes of the lysyl oxidase (LOX) and transglutaminase (TGM) family, stabilise fibrillar collagens in the extracellular space (98–101). In particular the enzymes LOX and LOX-like 2 (LOXL2) have been shown to crosslink fibrillar collagens both intra- and inter-molecularly (101) and LOXL2 has been put forward as a potential therapeutic drug target in IPF (102). Further collagen-associated proteins like the antioxidant proteins extracellular superoxide dismutase or glutathione peroxidase 3 (GPX3) may protect these long-lived molecules from oxidative damage (103–106).

## Collagen Turnover

In normal tissue homeostasis, the ECM is subject to a constant and dynamic, albeit typically slow, turnover, in which collagen is degraded and newly synthesised (107). The rate of collagen turnover differs drastically between tissue types. For instance, human cartilage collagen has a half-life of about 117 years, invertebral disc collagen 95 years, and skin collagen 15 years (108, 109). Even if, to our knowledge, the half-life of human lung collagen has not been determined, numerous studies have addressed lung collagen turnover in human and experimental animal lung tissue. Most studies point toward a remarkable level of constant *de novo* collagen synthesis and the presence of two collagen pools with different degradation rates; a pool of probably newly synthesised collagen which is subject to considerable degradation, and a pool of heavily crosslinked stable collagen which is more resistant to degradation (110–117). Considering the tightly wound triple-helical structure as well as the complex supramolecular structures described above, it is not surprising that only members of two protease families are capable of collagen degradation, namely MMPs and cathepsins. The so far described collagen degradation pathways can be categorised in extracellular and intracellular pathways. These processes are regarded as different from propeptide cleavage which is part of the normal collagen maturation pathway and reflects rates of *de novo* collagen synthesis rather than collagen turnover. Nevertheless, a strict distinction may represent an oversimplification. Notably, both processes can liberate biologically active peptide fragments, i.e., matricryptins or matrikines (57, 61).

### Extracellular Collagen Degradation

Extracellular collagen degradation is mainly attributed to **matrix metalloproteinases (MMPs)** and cathepsin K. MMPs are extracellular Proteolytic Zinc-dependent endopeptidases which collectively degrade all major ECM molecules. Collagenolytic activity has been observed for MMP1, MMP2, MMP7, MMP8, MMP9, MMP13, MMP14, and MMP19 (118, 119). MMP-mediated degradation of interstitial collagens, in particular of types I-III is best-described, but several MMPs (e.g., MMP2, MMP7, MMP9) also degrade basement membrane type IV collagen (118, 120–122). For a comprehensive overview of MMPs and their collagen substrates, the interested reader is referred to Visse and Nagase (123) and Jobin et al. (124). The C-terminal hemopexin domain of MMPs is crucial for collagen degradation as it not only recognises and binds the substrate but also unwinds the collagen structure in order to access the cleavage site (125, 126). In contrast, the cysteine protease **cathepsin K** (CatK) is probably the most effective protease for the degradation of extracellular fibrillar collagen (127, 128) because it can also target triple-helical collagen directly, without the need for unwinding of the helix (127).

### Uptake of Collagen and Intracellular Collagen Degradation

Uptake of collagen into the cell can occur by phagocytosis of an intact collagen fibril (129) or by macropinocytosis or receptor-mediated endocytosis of already cleaved collagen particles. In order to pass the cell membrane, collagen fibrils are recognised by integrins which triggers the process of phagocytosis (130). The so far observed integrins being involved are α1β1- and α2β1-integrin (131), as well as α10β1- and α11β1-integrins (132, 133). Initial fragmentation of fibrillar collagen is mediated by membrane-bound MMP14 (also termed MT-MMP1) (134).

Smaller collagen fragments, e.g., derived by extracellular MMP- or CatK-mediated degradation, can be taken up by two main pathways, macropinocytosis and receptor-mediated endosomal uptake. In macropinocytosis, solubilized collagen particles are internalised within actin-mediated endocytosis resulting in collagen-containing vacuoles (135, 136). Receptor-mediated endosomal uptake of collagen fragments is dependent on the urokinase plasminogen activator receptor-associated protein (uPARAP/Endo180 or C-type mannose receptor 2, MRC2). This endocytic mannose receptor mediates the internalisation of MMP-cleaved collagen fragments into clathrin-coated vesicles into fibroblasts (137–139). Receptors mediating endosomal collagen uptake in macrophages are macrophage mannose receptor 1 (MRC1) and lactadherin (MFGE8) (140, 141).

For intracellular collagen degradation, both pathways converge at the level of fusion with lysosomes. As to collagen-degrading proteases, lysosomes contain a range of **cathepsins**, including cathepsins B, D, K, and L, which cleave collagen into low-molecular-weight peptides (142–144). An overview of their known collagen substrates is given in Table 1. Notably, the same pathway can be used for newly synthesised collagen prior to secretion, for instance, when collagen is misfolded or otherwise “defective” (149, 150). In this so-called lysosome-dependent macroautophagy, lysosomes may fuse with ER- or Golgi-derived vesicles (151, 152).

**Table 1:** Cathepsins and their collagen substrates.

**Cathepsins**			
**Gene name**	**Protein name**	**Collagen substrate(s)**	**References**
CTSB	Cathepsin B	Collagen type IV	(145)
CTSD	Cathepsin D	Collagen type I	(146)
CTSK	Cathepsin K	Collagen type I and II	(143, 147, 148)
CTSL	Cathepsin L1	Collagen type I	(148)

*For MMPs, the reader is referred to excellent reviews by Visse and Nagase (123) and Jobin et al. (124), and references therein. Gene and protein nomenclature is according to UniProt*.

## Baseline Changes in Collagen Synthesis and Maturation During Mouse Lung Ageing

**Figure 4:**
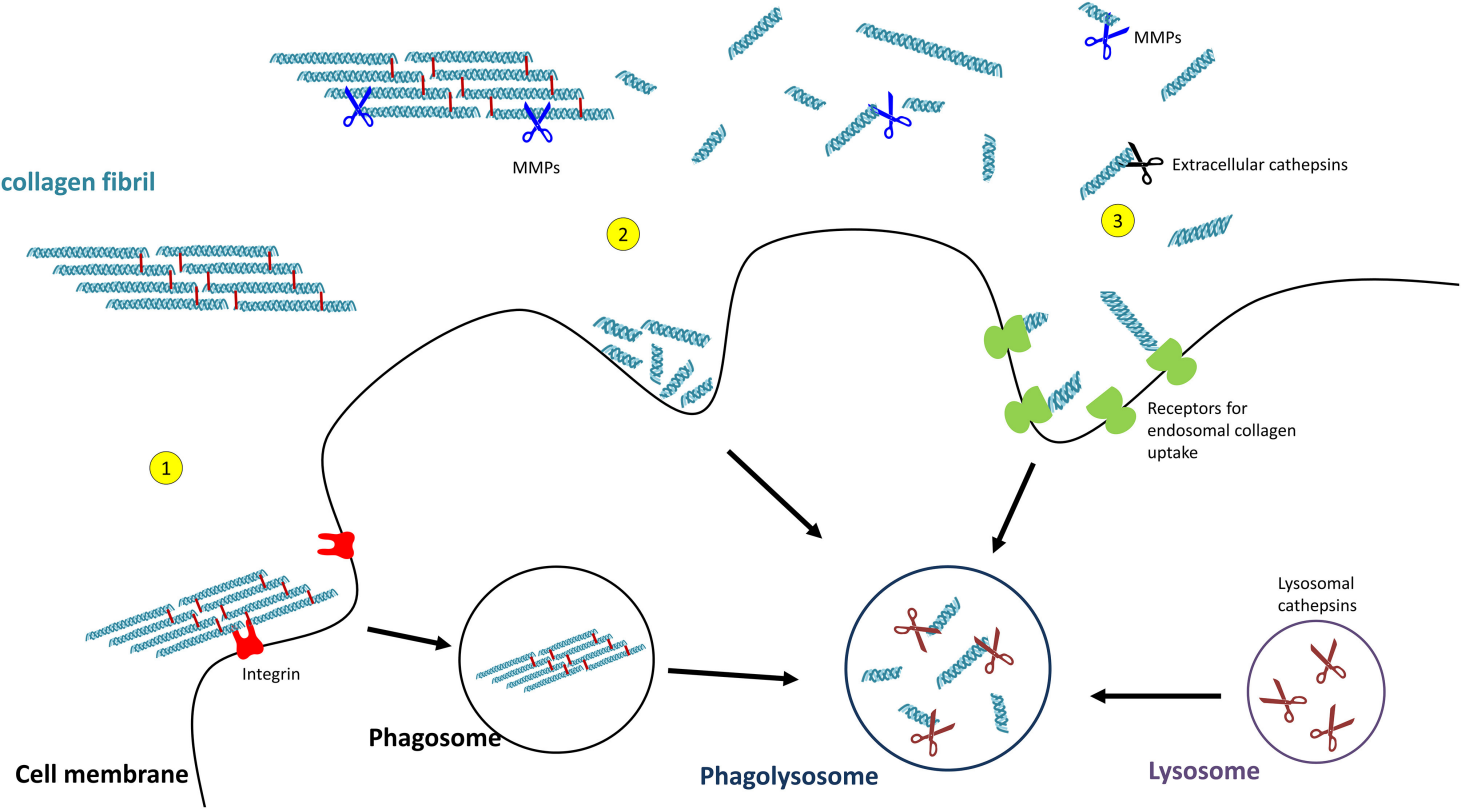
Pathways of collagen degradation. Both extracellular degradation by MMPs and extracellular cathepsins on the one hand and intracellular degradations by (1) collagen phagocytosis, (2) macropinocytosis, or (3) receptor-mediated endocytosis occurs.

**Figure 5:**
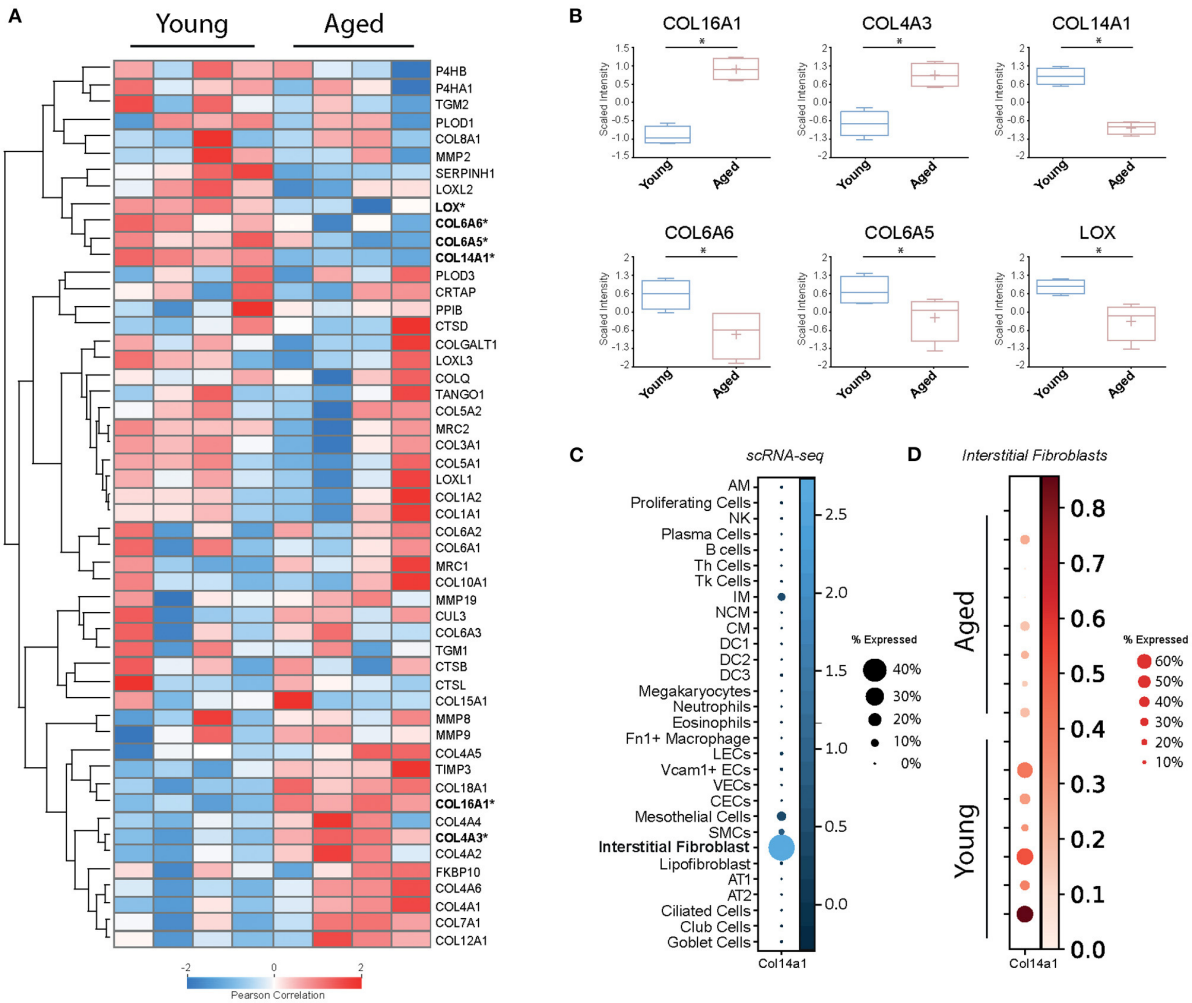
Baseline changes of proteins involved in collagen biosynthesis, trafficking, processing, and degradation. **(A)** Heatmap of normalised mean intensity values of 52 regulated extracellular matrix proteins (whole lung proteome, mass-spec) grouped by unsupervised hierarchical clustering and *Z*-scored (Pearson's correlation). **(B)** Boxplots of 6 significantly regulated proteins with age. **(C)** The dot plot shows mRNA expression specificity of Col14a1 in scRNA-seq data of whole lung homogenate. **(D)** Dotplot indicating mRNA expression levels between aged and young mice within the lung interstitial fibroblasts cluster (* = Student's *t*-test significant, FDR<10%).

In order to examine baseline changes, i.e., changes in the absence of environmental stimuli, in collagen biosynthesis, maturation and degradation (Figures 2, 4) upon ageing, we took advantage of a recently published single-cell RNA-Seq and multi-omics analysis data set on the ageing mouse lung (Figure 5, Table 2) (160). Following extraction of data for all relevant proteins, we observed no significant changes for proteins of the ER-resident machinery of collagen biosynthesis, modification, and triple helix formation (e.g., P4H and PLOD enzymes, SERPINH1, FKBP10, PPIB, Figure 5A). In contrast, we found expression of several collagen subtypes deregulated upon normal ageing: The type VI collagen chains COL6A5 and COL6A6, as well as COL14A1, were significantly downregulated, while levels of COL4A3 and COL16A1 were significantly increased. Furthermore, levels of the collagen crosslinking enzyme lysyl oxidase (LOX) were decreased (Figures 5A,B). Table 2 provides a summary of these findings including a comparison with what is currently known about corresponding changes in human tissues upon ageing.

**Table 2:** Overview on collagens and collagen biosynthetic proteins observed to be regulated with age in the mouse lung.

**Collagen**	**FC^**a**^ (old/young) mouse lung**	**major expressing cell type(s)^**b**^**	**Altered in human ageing tissue?**	**Relevance to human disease**
COL4A3	+2.9	AT1	No evidence for COL4A3 COL4A2 ↓ with age in adipose tissue COL4A5 ↑ with age in skin COL4A6 ↑ with age in skin (153)	Goodpasture syndrome (154) Alport syndrome (155)
COL6A4	−15	Not enough data	Pseudogene	Unknown
COL6A5	−3.9	(Myo)fibroblasts, pericytes, smooth muscle cells	No evidence	Unknown
COL6A6	−3.4	(Myo)fibroblasts, smooth muscle cells	No evidence	Unknown
COL14A1	−2.7	Fibroblasts, pericytes, smooth muscle cells	↓ with age in skin (153)	Missense mutation causes punctate palmoplantar keratoderma (156, 157)
COL16A1	+19	Fibroblasts, basal cells, ciliated cells	↑ with age in skin (153)	Unknown
LOX	−4.6	Fibroblasts, mesothelial cells	↓ with age in skin (153)	Loss of functions mutation in *LOX* predispose to thoracic aortic aneurysms, dissections, and ruptures (158, 159)

a *FC, Fold Change, extracted from (160)*.

b *According to recent scRNA-Seq data (160–164)*.

*Evidence for similar change in human tissues and relevance of these genes and proteins for human disease*.

### Downregulation of Type VI Collagen Chains COL6A5, COL6A6

Type VI collagen differs from most other collagens by its unique supramolecular assembly, the formation of characteristic beaded microfilaments in the ECM, a property which it only shares with type XXVI (COL26A1) and type XXVIII collagen (COL28A1) (23) (Figure 1). Type VI collagen is a component of the basement membrane in lung, muscle and skin (165), but can also localise to the pericellular matrix, e.g., in tendon (166, 167). A number of studies, including our own, indicate an important role of type VI collagen for adhesion, migration, proliferation, death, and dysfunction of adherent cells (77, 166–174). Typically, type VI collagen consists of the α1(VI), α2(VI), and α3(VI) chains, which assemble in a 1:1:1 ratio and are encoded by *Col6a1, Col6a2*, and *Col6a3*, respectively (166). These three chains are found in all connective tissues, they are by far the most abundant type VI collagen chains, including in the lung, and their levels are not altered during lung ageing in the mouse (160).

Expression of the comparatively recently discovered type VI collagen chains α4(VI), α5(VI), and α6(VI), in contrast, is more restricted, but at least two of these chains are consistently expressed in foetal, new-born, and adult lung (28, 175). An important difference between the murine and human genes is that the human *COL6A4* gene is disrupted and not functional. In mice, expression of *Col6a4* is mostly observed in new-born tissue—including lung—but lost in all adult tissues except for uterus and ovaries (28, 175, 176). The complete loss of COL6A4 from young to old mouse lungs may therefore correspond to remnant *Col6a4* from developmental expression, which is increasingly degraded during normal collagen turnover in a mouse's lifetime, becoming undetectable in the old lung (160). In contrast, *COL6A5* and *COL6A6* are both expressed in adult human lung, even at higher levels than in foetal lung, arguing for a role in adult lung function (28).

Based on sequence similarities, co-purification and colocalization analysis, both COL6A5 and COL6A6 are predicted to assemble with COL6A1 and COL6A2 as an alternative chain to COL6A3 (28, 175, 177). However, strong biochemical evidence in support of this hypothesis is still lacking. Even though some of the α5(VI) (COL6A5) and α6(VI) (COL6A6) chains may be engaged in type VI collagen triple helix formation, in the lung the α3(VI) chain is more abundant by several orders of magnitude, and expression of *Col6a3* is unchanged during ageing. Thus, an α5/α6(VI) -> α3(VI) chain switch from young to old lung tissue is unlikely to represent a major event affecting the general function of type VI collagen. Interestingly, type VI collagen, in particular the α3(VI) chain (COL6A3), has been attributed a role in stemness and promotion of tumour growth. Therefore, local replacement of α5/α6(VI) by α3(VI) chains during ageing may modulate stem cell niches, maybe even contribute to stem cell exhaustion, a major hallmark of ageing (178, 179). But also presence of non-triple-helical (NTH) COL6A5 and COL6A6 polypeptides is conceivable, as evidence for alternative NTH collagen gene products is emerging in the literature, e.g., for COL6A1 (22, 180) and COL4A1 (181, 182).

### Downregulation of Type XIV Collagen (COL14A1)

Type XIV collagen belongs to the FACITs (Figure 1) and plays an important role in fibrillogenesis of type I collagen, in particular during development (183–187). Similar to downregulation of COL14A1 in the ageing mouse lung, downregulation of COL14A1 has also been described for human skin ageing (153). Several studies support a role of COL14A1 in connecting the basement membrane to the subepithelial interstitial matrix (185, 188, 189). Like COL6A4 mentioned above, COL14A1 is developmentally regulated in some tissues, e.g., in tendon or the heart (189–192).

Interestingly, several reports in the past have independently argued for an important role of COL14A1 in the maintenance of normal epithelial cell turnover and differentiation in adult tissues. For instance, a missense mutation in COL14A1 at a highly conserved amino acid residue (Pro1502Leu) leads to punctate palmoplantar keratoderma, a skin disease characterised by aberrant squamous cell differentiation leading to hyperkeratosis in the cornified layer (156, 157). In renal cell carcinoma (RCC), which arises from epithelial cells, frequent hypermethylation of COL14A1 has been observed which resulted in transcriptional silencing; knockdown of COL14A1 increased the growth rate of RCC cell lines (193). COL14A1 is also notably downregulated in oesophageal squamous cell carcinoma (194). Collectively, these observations point toward an anti-proliferative or tumour suppressor function of COL14A1.

With this documented role on epithelial cell survival and differentiation, it is at first glance surprising that recent single-cell RNA-Seq data consistently show expression of COL14A1 by interstitial fibroblasts, not epithelial cells (160–164) (Figure 5C). A study on fibroblast heterogeneity even revealed that expression of COL14A1 marks a specific matrix-producing fibroblast subtype which increases in cell number in murine lung fibrosis (195). As lung fibrosis is characterised by loss of normal alveolar type I and type II cells and atypical epithelial differentiation (196), it may be speculated that overexpression and extracellular deposition of fibroblast-generated COL14A1 contributes to these epithelial events and therefore to loss of normal alveolar structure. In addition, loss of Col14a1 in the ageing mouse lung correlates with changes in cell type composition in mouse airway epithelial cells (160). Overall, it seems that deregulation or loss of paracrine COL14A1 has profound consequences on epithelial survival and differentiation and may therefore contribute to the observed changes during ageing. These observations warrant future studies on the exact distribution and molecular function of COL14A1 in lung ageing and disease.

### Upregulation of Type IV Collagen (COL4A3)

Type IV collagen is a major structural component of the basement membrane and represents the prototypical network-forming collagen. Six genes (Col4a1-Col4a6) encode for six distinct type IV collagen chains which can assemble into three heterotrimeric molecular isoforms, namely α1(IV)_2_α2(IV), α3(IV)α4(IV)α5(IV), and α5(IV)_2_α6(IV) (197). At least the first two heterotrimeric forms have been reported in the lung, the major one being α1_2_α2(IV) (22, 198). Interestingly, Angelidis et al. (160) found that, even if only COL4A3 passed the threshold for significance, protein levels of all six type IV collagen chains were increased in the ageing lung. These changes went in parallel with alterations of other basement membrane-specific proteins, like decrease of FRAS1, FREM1, FREM2, and COL14A1 discussed above. Intriguingly, the authors also found that transcript levels for all type IV collagen genes anti-correlated with protein levels, i.e., were downregulated with ageing. While downregulation of type IV collagen chain transcripts occurs consistently upon ageing of human tissue and cells (153, 199, 200), the post-transcriptional mechanisms that control levels of type IV collagen protein may be tissue-specific: While, similar to the results in ageing mouse lung, Karttunen et al. reported type IV collagen protein to be increased in human kidney upon ageing (201), in skin, type IV collagen protein was found to decrease upon ageing, in parallel to transcript levels (200). Interestingly, a decrease of serum type IV collagen has been described upon ageing (202) which may in part reflect increased type IV collagen retention and deposition in tissue. Overall, type IV collagen turnover, however, as measured by the formation product P4NP7S and the degradation product C4M in serum, seems to be stable during ageing (203).

### Upregulation of Type XVI Collagen (COL16A1)

Just like type XIV collagen, also type XVI collagen belongs to the FACIT family of collagens and forms homotrimeric triple helices (23, 204). It is a minor collagen component which shows a tissue-dependent versatility for incorporation into different collagen suprastructures (205, 206). Its distribution in lung ECM is unknown, but judging from recent scRNA-Seq data, where COL16A1 is found expressed by all fibroblast subtypes as well as a broad range of epithelial cells, it is likely to be a constituent of both the basement membrane and the interstitial matrix (160–164). Even though COL16A1 has been observed to be increased upon ageing also in human skin (153), its potential role in the ageing process has not received much attention. Tajima et al. found that increased COL16A1 expression in dermal fibroblasts correlated with cell growth arrest of these cells (207). In contrast, induction of COL16A1 expression promotes proliferation and invasion of cancer cells (205, 208–210). Similar to our speculations on COL14A1, there may be analogous paracrine effects by fibroblast-generated COL16A1, albeit in reverse directions: Loss of fibroblast-generated COL14A1 on the one hand, as well as induction of COL16A1 deposition by fibroblasts on the other, may lead to increased cell proliferation and aberrant differentiation of adjacent epithelial cells and thus contribute to tumorigenesis and tumour invasion. Notably, this is reminiscent of a proposed link between ageing and cancer where senescent fibroblasts secrete factors, including extracellular matrix components that promote epithelial tumorigenesis (211, 212). Clearly, future studies are needed to decipher the role of COL14A1 and COL16A1 in this context.

### Downregulation of Lysyl Oxidase

Lysyl oxidase (LOX) is a copper-dependent protein-lysine-6-oxidase, activity of which is critical for stabilisation of extracellular fibrillar collagen (71, 213). During fibril formation, LOX catalyses the oxidative deamination of specific lysine and hydroxylysine residues in the N- and C-telopeptides of fibrillar collagens, resulting in the corresponding aldehyde forms (71) (Figure 3B). These reactive intermediates trigger a series of subsequent condensation reactions between triple-helical subunits of collagen fibres, ultimately leading to divalent or trivalent crosslinks.

Several studies have shown that downregulation of LOX in the context of ageing is not restricted to the lung. For instance, *LOX* expression is reduced with age in human, rat and monkey skin (153, 214, 215), in urogenital tissues of female mice (216) and in rat aorta (217). Notably, loss of function mutations in *LOX* predispose to aortic aneurysms and age is the most important risk factor for aortic aneurysms (158, 159), supporting the concept that loss of LOX upon ageing may have direct effects on ECM integrity and tissue stiffness. Downregulation of LOX upon ageing appears to correlate with fewer reducible difunctional LOX-derived crosslinks in skin, aorta and lung (218, 219), even though, to our knowledge, no study has assessed this correlation directly. This contrasts with non-enzymatic collagen crosslinking which increases with age and will be discussed in more detail below (220). Overall, also taking into account recent findings that the same collagen lysines are targeted by LOX-mediated and non-enzymatic collagen crosslinking events (221), there seems to be a shift from LOX-mediated to advanced-glycation end product (AGE) crosslinks upon ageing.

In cancer research, LOX has received considerable attention, both owing to tumour growth and invasion-promoting properties on the one hand and to tumour suppressor functions on the other hand (222, 223). LOX is synthesised as a pro-enzyme and activation requires removal of the pro-peptide, notably by the same enzymes that cleave off the C-terminal pro-peptide of type I collagen (BMP1/Tolloid-like proteinases) (71). The mature protein is overexpressed in various cancer types including lung adenocarcinoma, typically correlates with poor prognosis, and has been described to create a stiffer microenvironment which supports tumour growth and metastasis (222, 224–230). In contrast, the tumour-suppressing activities of LOX are attributed to the released pro-peptide (222, 223, 231, 232).

### The Impact of Ageing on Collagen Degradation

Few studies have directly assessed changes in collagen synthesis and degradation upon ageing. Several early studies, measuring the collagen synthesis rate in tissues of young and aged experimental animals, including lungs, as well as *ex vivo* culture of human skin biopsies, consistently found evidence for a decreased synthesis rate with ageing tissue (233–235). Mays et al., in addition, studied how much of the newly synthesised collagen is degraded and found that, in aged rats, a larger percentage was directly subjected to degradation than in young rats (233). Thus, in older age not only less collagen would be synthesised, but also newly synthesised collagen would be more prone to direct degradation. This concept, however, has been challenged by a recent study on old *vs*. young mouse lungs where the authors demonstrate that aged mice have higher overall levels of total collagen and lower levels of a collagen endocytic receptor termed mannose receptor, C-type 2 (MRC2), the major receptor for fibroblast-mediated intracellular degradation of collagen (236). Notably, in the proteomics data set presented here, we found lower levels of MRC2 in three out of four aged animals, albeit without reaching statistical significance (Figure 5A), which may reflect the limited statistical power of the proteomics data set. Furthermore, levels of most major lung collagens (types I, III, IV) remained unchanged or were even increased in old *vs*. young mouse lungs (Figure 5) (160). Overall, this indicates that mature collagen is very stable in the adult lung.

Partially supporting this concept, we observed no consistent changes for collagen-degrading proteases in the data set on mouse lung ageing presented here (Figure 5A) (160). Neither levels of the detected MMPs and TIMPs (MMP2, MMP8, MMP9, MMP19, TIMP3) nor levels of the detected cathepsins (CTSB, CTSD, CTSL) were significantly altered (160). Of course, this is only circumstantial evidence as (a) this study did not provide a comprehensive quantification of all lung collagenases and their inhibitors, (b) levels *per se* cannot be equated with enzymatic activity, and (c) accessibility of cleavage sites in the collagen substrate itself can be masked or altered upon ageing. As to enzymatic MMP activity, Calabresi et al. have shown that natural ageing of rat lungs is accompanied by decreases in MMP1 and MMP2 activity in parallel with moderately increased levels of the MMP inhibitors TIMP1 and TIMP2 levels (237), suggesting even a loss of collagenase activity upon ageing. Furthermore, and maybe even more importantly, there is evidence suggesting that the degradability of collagen is affected by ageing-related collagen modification and crosslinking (e.g., non-enzymatic crosslinking by advanced glycation end-products, AGEs, discussed below) and addition or removal of glycosaminoglycans (238–243). Thus, protease activity by itself may not be the decisive factor after all, but in fact the accessibility of the corresponding collagen cleavage sites.

It is to date unclear, whether these findings reflect the human situation. Collagen degradation upon ageing in the human lung has not been directly assessed. A number of studies have measured circulating levels of MMPs and TIMPs and deduced changes in ECM turnover upon ageing. However, the reported findings are not consistent (244–248) and even if such changes may be of importance for collagen degradation within the vascular wall, it is unclear how peripheral levels of MMP and TIMPs correlate with the integrity of the ECM in other tissues. Obviously, tissue-resident levels and activities of proteases in correlation with ECM integrity of the studied human tissue are more relevant to examine in that context, but such studies are much scarcer. An immunohistochemical study of human lungs found an age-dependent increase of TIMP2^+^ cells, but not MMP2^+^ cells, in the lung (249), providing some evidence for decreased MMP-mediated collagen degradation in the aged lung. Notably, in a recent pioneering study of human skin ageing, the authors correlated matrisome changes with the assessment of ECM integrity by single harmonic generation and two-photon autofluorescence imaging (214). Among other changes, they found decreased MMP2 and increased TIMP3 levels in aged skin. The mechanisms underlying skin ageing, however, involve environmental insults, e.g., exposure to UV light, microorganisms, and mechanical stress that do not apply to the lung.

Undoubtedly, more research is warranted to elucidate mechanisms of collagen and ECM degradation upon ageing of the lung. Nevertheless, taken together, the current evidence supports an age-dependent decline of lung collagen degradation, suggesting that, also in the lung, collagen is a long-lived molecule and thus susceptible to the accumulation of damage with time.

## Non-enzymatic Collagen Crosslinking—Advanced Glycation Endproducts

**Figure 6:**
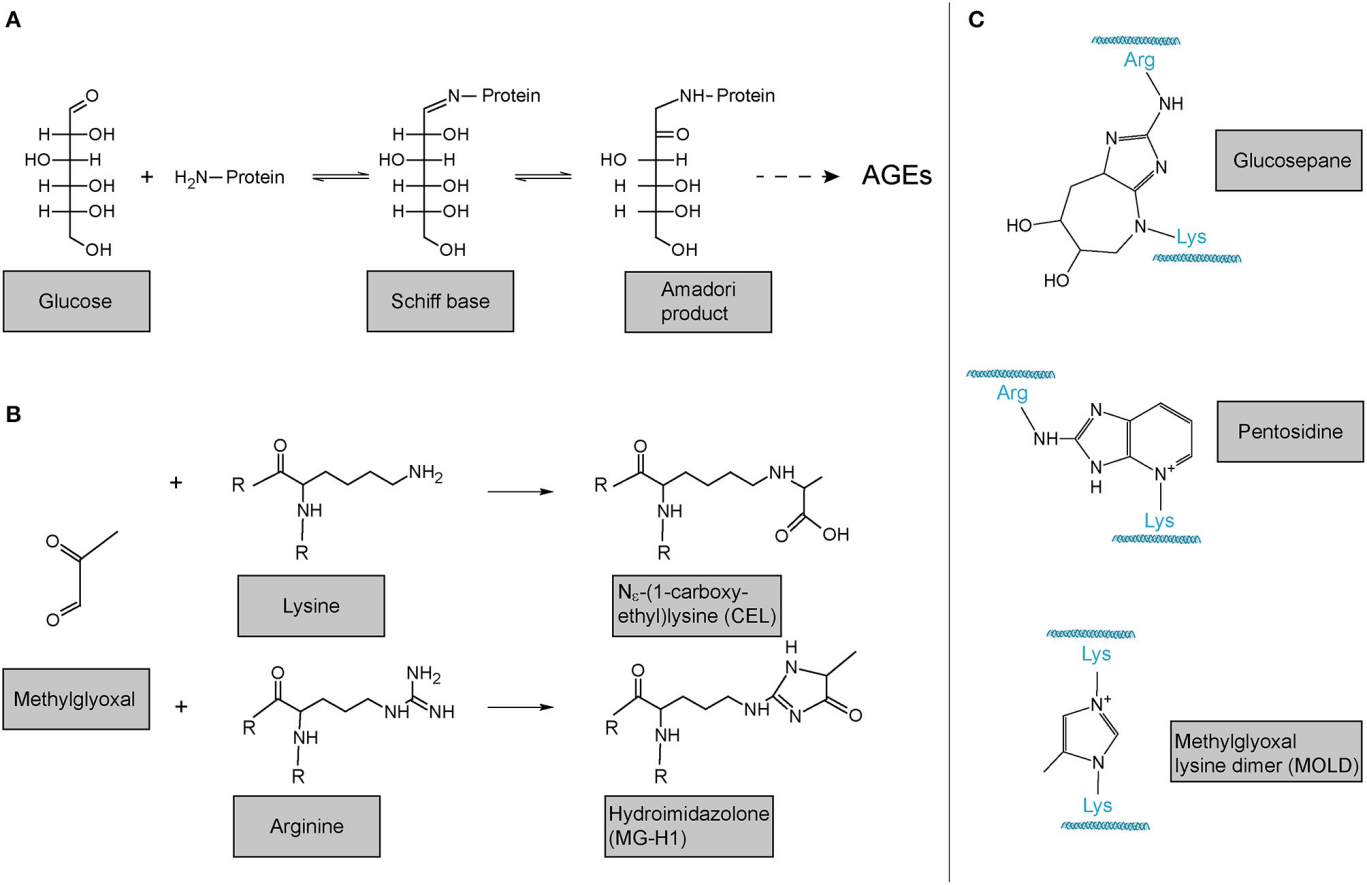
Advanced glycation endproducts (AGE)-mediated collagen modification and crosslinking. **(A)** In the classical Maillard reaction, a reducing sugar (here: glucose) reacts with an amino group in a protein, e.g., the protein's amino terminus or amino groups of the side chains arginine or lysine. The resulting Schiff base rearranges to a ketoamine, the so-called Amadori product. Subsequent reactions of this initial product result in the irreversible formation of different protein adducts and crosslinks, collectively termed advanced glycation endproducts (AGEs). **(B)** Reactions of the dicarbonyl methylglyoxal with protein lysines or arginines results in the formation of protein adducts as e.g., N_ε_-(1-carboxyethyl)lysine (CEL) or the hydroimidazolone MG-H1. **(C)** Structures of some of the most common AGE-mediated crosslinks. Structures were generated using ACD/Chemsketch freeware.

In addition to LOX- and TGM-mediated, i.e., enzymatic crosslinking mentioned above, also non-enzymatic crosslinking of collagen occurs. The probably most important one in the context of human disease and ageing is the reaction of collagen with sugars or sugar metabolites which results in the formation of so-called advanced glycation endproducts (AGEs). These products are principally not restricted to collagen. In the best-described classical Maillard reaction, any free amino group, e.g., the ?-amino group of a lysine or the protein N-terminus, reacts with the carbonyl group of a reducing sugar to form a Schiff base. This unstable Schiff base is spontaneously rearranged into a more stable keto amine intermediate, the so-called Amadori product (Figure 6A). This early glycation product is in equilibrium with glucose and still reversible. However, a small part of these Amadori products undergo subsequent, predominantly oxidative, events resulting in the irreversible formation of different protein adducts and protein crosslinks, collectively termed AGEs. As the latter reactions occur over months and years, such adducts and crosslinks accumulate in long-lived proteins like extracellular collagens, including in the lung, and strongly correlate with age (220, 250–253). Figure 6A shows the classical Maillard reaction and some of the best-characterised and most often analysed AGEs are depicted in Figure 6B [N_ε_-(1-carboxyethyl)lysine, CEL; hydroimidazolone, MG-H1] and Figure 6C (lysine-arginine cross-links pentosidine and glucosepane; methylglyoxal lysine dimer, MOLD).

Notably, while the above-described reactions of proteins with reducing sugars are the best-described, AGEs can also be formed by the reaction of lysines and arginines with other carbonyl compounds, in particular highly reactive dicarbonyl compounds (254, 255). Accumulation of such compounds and its consequences is also referred to as “dicarbonyl stress” in the literature. Methylglyoxal (Figure 6B) is an abundant representative of these dicarbonyl compounds. Methylglyoxal is generated during the formation of AGEs in the context of the above-described glycation of proteins by reducing sugars, but also, in fact more importantly, endogenously as a result of spontaneous degradation of triosephosphates, degradation of glycated proteins, oxidation of aminoacetone in threonine catabolism, and peroxidation of lipids (256–258). In the glycation reaction (Figure 6B), methylglyoxal is about 20.000 times more reactive than glucose. It is thus not surprising that an enzymatic detoxification system has evolved to control intracellular methylglyoxal levels: The glyoxalase system, which consists of the enzymes glyoxalase 1 and 2 (Glo1 and Glo2) and a catalytic amount of reduced glutathione, catalyses the formation of lactate from methylglyoxal (256–259). Interestingly, evidence is emerging that methylglyoxal plays an important role in ageing and age-related disease: In the nematode *Caenorhabditis elegans*, a model organism frequently used in fundamental research on ageing and lifespan-extending mechanisms, glyoxalase activity is markedly reduced upon ageing and overexpression of the *C. elegans* orthologue of Glo1 increased lifespan (260, 261). Glyoxalase expression and/or activity has been reported to decrease upon ageing in human arterial tissue (262), in red blood cells (263), in human brain (256, 264), as well as in mouse lung (160). Methylglyoxal-protein adducts are increased in ageing human lens and skin (265, 266). Also, studies in rats have shown that overexpression of Glo1 protects from age-related renal dysfunction (267). Importantly, even if generated intracellularly, methylglyoxal can easily target the extracellular matrix because it is freely membrane-permeable (268) and has a relatively long range of diffusion—its tissue levels are therefore critically dependent on local intracellular glyoxalase activity (257). In summary, it is highly plausible that methylglyoxal and the glyoxalase system also contribute to molecular alterations of the ECM and to lung dysfunction upon ageing, even though direct evidence for this is lacking to this day.

Identification and quantification of AGEs are difficult tasks. First of all, AGEs are derived from many different precursor molecules and represent a highly heterogeneous group of compounds (253, 269). New variants of AGEs are still being discovered (270) and it is likely that not all AGE-protein adducts and crosslinks are known in molecular detail. For the analysis of clinical samples, early detection methods took advantage of the fluorescent properties of many AGEs: Collagen from connective tissue was extracted and solubilized by collagenase digestion and fluorescence measured [≈370 nm excitation/440 nm emission (271)]. More recently, autofluorescence of non-pigmented skin has been shown to correlate with collagen-linked fluorescence, concentrations of the fluorescent AGE pentosidine (Figure 6C), concentrations of the non-fluorescent AGEs N_ε_-(1-carboxymethyl)lysine (CML) and N_ε_-(1-carboxyethyl)lysine (CEL, Figure 6B), as well as with age, diabetes duration, and diabetic complications (272–278). Even though clearly a convenient non-invasive technique, any technique based on fluorescence will be limited in specificity and sensitivity, as not all AGEs are fluorescent including major representatives like CML and CEL and interference by other fluorescent molecules can occur, e.g., by NAD(P)H. Numerous attempts have been made to establish immunochemical detection of AGEs in tissue, albeit yielding inconsistent results due to lack of specificity, sample processing artefacts, and contamination by glycated blocking proteins (279–282). Therefore, quantitative results obtained by enzyme-linked immunosorbent assays (ELISAs), for instance, must be interpreted with caution.

The state-of-the-art detection method for AGEs in tissue and clinical samples is high performance or ultra-high performance liquid chromatography combined with mass spectrometry (279). New trending approaches, collectively referred to as “Maillard proteomics” are expected to revolutionise our understanding of glycation site specificity of AGE adducts, a prerequisite to gain an understanding of their function (269).

Even though the association between increases in AGE-mediated crosslinking, increased collagen stiffness, and decreased solubility with ageing is well-established (251), to the best of our knowledge surprisingly few studies have assessed AGEs and collagen AGE adducts in the lung. This may reflect the technical difficulties of specific AGE quantification pointed out above. Nevertheless, measurements of AGE-related fluorescence in the context of two animal studies support the concept that also in lung collagen AGE-load increases with age and anti-correlates with enzymatic solubility (220, 283). Interestingly, a study using the bleomycin-induced mouse model of lung fibrosis suggests that inhibition of AGE formation may protect from lung fibrosis (284). In reverse, accumulation of AGE load in the lung upon ageing may therefore represent one of the reasons why idiopathic lung fibrosis predominantly occurs in the elderly. Resonating with this concept, several reports indicate that diabetes mellitus, where increased blood glucose levels lead to increased tissue levels of AGEs, increases the risk for pulmonary fibrosis (285–288).

### The Consequences of “AGEd” Collagen

But what can be the consequences of non-enzymatically glycated—or “AGEd”—collagen? There is evidence that AGEs increase collagen fibril stiffness and attenuate collagen turnover by, on the one hand, inhibiting degradation by MMPs and cathepsin K (239, 243, 289), and, on the other hand, inhibiting phagocytosis pathways (290). Furthermore, experiments with *in vitro* generated AGE-crosslinked collagen, by e.g., incubating collagen with ribose or methylglyoxal and measuring mechanical properties, indicate that in particular molecular sliding of collagen fibrils and fibres is affected by this type of crosslinking (291, 292). Hence, most likely “AGEd” fibrillar collagen contributes to the generally observed AGE-induced reduction of viscoelasticity of connective tissues accompanied by increased mechanical fragility (254, 291, 293–295).

In addition to changes in collagen turnover and biomechanics, AGE-mediated modification and crosslinking of collagen has the potential to mask important collagen-cell or collagen-protein-interaction sites with direct effects on adherent cell behaviour and function. Indeed, several studies with diverse cell types support this concept. For instance, non-enzymatically glycated collagen exhibits reduced affinity to heparin and keratan sulphate proteoglycans, resulting in diminished adhesion of B cells and reduced migration of endothelial cells (296). Similarly, methylglyoxal-modified type IV collagen displays reduced affinity to integrins, leading to attenuated adhesion of renal glomerular cells, and reduced attachment, viability, and angiogenic activity of endothelial cells (297, 298). Also neurons cultured on glycated type I collagen, show altered morphology and function in comparison to culture on normal type I collagen including reduced neuron interconnection and increased release of pro-inflammatory stimuli like nitrite and TNF-α (299). Finally, it has been reported that AGE-modified collagen fails to interact with discoidin domain receptor 2 and thus loses the capacity to upregulate expression of LOX in osteoblasts (300). Notably, even if direct evidence is lacking, this provides a potential mechanism underlying the above-described loss of LOX upon ageing in the lung and highlights a potential direct relationship between accumulation of AGE-mediated modifications/crosslinks and downregulation of LOX-mediated crosslinks.

Interestingly, molecular modelling studies using a collagen type I microfibril have put forward a number of candidate inter- and intramolecular Lys-Arg pairs that fulfil the requirements in terms of configuration and distance (301) and change in enthalpy (302) to allow for the formation of glucosepane crosslinks. In both studies, the authors identified more potentially AGE-crosslinked arginines and lysines within binding sites for integrins, proteoglycans, MMPs and other proteins. Collectively, these findings emphasise that “AGEd” collagen has a major impact on adherent cell function in many different tissues and clearly contributes to disease, warranting similar studies in the lung.

### Effects Mediated Through the Receptor for Advanced Glycation Endproducts

AGEs are ligands for the receptor for advanced glycation endproducts (RAGE), a membrane-bound receptor of the immunoglobulin family (303, 304). Originally discovered in 1992 as an AGE-binding receptor (305), several additional ligands have been identified including S100/calgranulins, amyloid fibrils, amphoterins, and Mac-1. RAGE can thus be viewed as a relatively promiscuous pro-inflammatory pattern recognition receptor (303, 304). RAGE is abundantly expressed in the lung, at baseline predominantly in alveolar type I cells toward the basal membrane, but it can be activated in many other cell types upon exposure to RAGE ligands (304). Numerous studies have established associations of RAGE and its soluble decoy receptor sRAGE with lung injury and disease (303, 304, 306, 307).

RAGE-mediated downstream signalling depends on the identity and oligomerization state of the ligand and the tissue context. Ligand-engaged RAGE can trigger oxidative stress and multiple signalling cascades including p21 ras, erk1/2 (p44/p42) MAP kinases, p38 and SAPK/JNK MAP kinases, rho GTPases, phosphoinositol-3 kinase, and the JAK/STAT pathways. This leads predominantly to sustained activation of NF-κB- and STAT-dependent gene transcription (304, 308–310). As, typically, studies have been performed using soluble and globular glycated proteins such as serum albumin or ovalbumin (305, 311–313), it is not entirely clear whether “AGEd” collagen is capable of inducing these pathways. Studies using glycated collagen have established a RAGE-dependent link between “AGEd” collagen and apoptosis of mesenchymal cells. For instance, N_ϵ_(1-carboxymethyl) lysine (CML)-collagen (derived from reaction of collagen with glyoxal instead of methylglyoxal, Figure 6B), but not control collagen, induces fibroblast and osteoblast apoptosis *via* the RAGE/p38/JNK axis, but largely independent of NF-κB signalling (314, 315). Interestingly, some evidence indicates that RAGE promotes adhesion to collagen without the need for AGE-mediated modification, even though the glycation status of the collagen used was not reported in these studies (316, 317).

## Conclusions

There can be no doubt that collagen plays an important role in lung physiology and pathology. Even though the collagen family of proteins has been the subject of studies for many decades it is striking how little we still know about collagen composition changes and molecular alterations in lung ageing and disease. Here, we provided a comprehensive review on mechanisms of collagen biosynthesis, processing, modifications and crosslinking and how these change upon ageing. Collectively, a picture emerges where ageing (1) leaves the normal ER-resident collagen biosynthesis machinery largely unaffected, but (2) results in distinct changes in collagen composition and (3) changes in the molecular nature of collagen crosslinks. Clearly, these observations warrant future work to address the mechanistic consequences of these changes in the context of lung ageing and disease. Ultimately, such studies may be critical to the field of lung regenerative medicine.

## Author Contributions

CO, ED, and CS-W wrote the manuscript and prepared figures. HS and IA provided data on lung ageing and prepared Figure 5. All authors proofread and edited the manuscript.

## Conflict of Interest

The authors declare that the research was conducted in the absence of any commercial or financial relationships that could be construed as a potential conflict of interest.
